# Microvilli Adhesion: An Alternative Route for Nanoparticle Cell Internalization

**DOI:** 10.1021/acsnano.1c03151

**Published:** 2021-09-29

**Authors:** Patrizia Sommi, Agostina Vitali, Stefania Coniglio, Daniele Callegari, Sofia Barbieri, Alberto Casu, Andrea Falqui, Lorenzo Vigano’, Barbara Vigani, Franca Ferrari, Umberto Anselmi-Tamburini

**Affiliations:** † Human Physiology Unit, Department of Molecular Medicine, University of Pavia, Via Forlanini 6, 27100 Pavia, Italy; ‡ Department of Chemistry, University of Pavia, 27100 Pavia, Italy; § Department of Physics, University of Pavia, 27100 Pavia, Italy; ∥ Biological and Environmental Sciences and Engineering Division, NABLA Lab, King Abdullah University of Science and Technology (KAUST), 23955-6900 Thuwal, Saudi Arabia; ⊥ Department of Drug Sciences, University of Pavia, 27100 Pavia, Italy

**Keywords:** CeO_2_, nanoparticles, HeLa, adhesion/internalization, cell microvilli

## Abstract

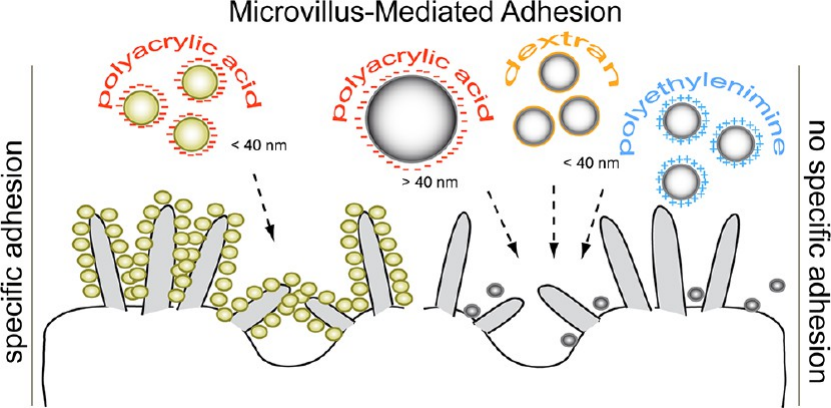

The cellular uptake of nanoparticles (NPs) represents a critical step in nanomedicine and a crucial point for understanding the interaction of nanomaterials with biological systems. No specific mechanism of uptake has been identified so far, as the NPs are generally incorporated by the cells through one of the few well-known endocytotic mechanisms. Here, an alternative internalization route mediated by microvilli adhesion is demonstrated. This microvillus-mediated adhesion (MMA) has been observed using ceria and magnetite NPs with a dimension of <40 nm functionalized with polyacrylic acid but not using NPs with a neutral or positive functionalization. Such an adhesion was not cell specific, as it was demonstrated in three different cell lines. MMA was also reduced by modifications of the microvillus lipid rafts, obtained by depleting cholesterol and altering synthesis of sphingolipids. We found a direct relationship between MAA, cell cycle, and density of microvilli. The evidence suggests that MMA differs from the commonly described uptake mechanisms and might represent an interesting alternative approach for selective NP delivery.

The cellular internalization of nanoparticles (NPs) has been extensively investigated in the past two decades to exploit the potential benefits of NPs or to counteract their harmful effects on biological systems. NPs can, due to their small size (<100 nm), cross physiological barriers such as skin, blood–brain barrier, and gastric mucosal barrier and then interact directly with underlying tissues at the cellular level.^1−7^ These characteristics make them potentially harmful but also offer an opportunity for applications in therapeutics, drug delivery, and imaging. The relevance of these applications for medical treatment of significant diseases means that the study of the basic mechanisms involved in NP uptake and processing is still of interest.

The literature on NP–cell interaction varies widely due to significant differences in protocols, cell lines, and NP characteristics. However, it is largely recognized that the physicochemical properties of NPs play an essential role in making them more or less reactive toward the biological environment. Thus, size, stiffness, surface charge, functionalization, and composition, as well as the formation of a protein corona, play a pivotal role in this regard.^8−12^ Surface chemistry is especially relevant, and both surface coating and charge appear to be fundamental in determining if and how NP–cell interaction occurs. NPs are usually coated with polymeric macromolecules, such as polyethylene glycol (PEG), polyacrylic acid (PAA) or dextran to avoid aggregation and precipitation in the biological environment.^9,13,14^ These surface modifications have a profound influence on the interaction of NPs with cells and have been shown to have a pivotal influence on NP uptake and intracellular localization, as well as toxicity after internalization.^9^ It has been reported, for example, that cationic NPs are internalized more efficiently than neutral and anionic ones.^15^ NP dimensions also play an essential role in determining their uptake mechanisms.^8,10,16^ Theoretical analysis, supported by experimental evidence, has indicated that the optimal size for NPs to be efficiently internalized by cells is around 20–30 nm.^8,17−20^

In addition to the physicochemical characteristics of NPs, the status of the recipient cell can also influence the level of internalization.^21,22^ Kim and colleagues observed that while cells in different phases internalize NPs similarly, at the end of one complete cell cycle they show a different level of NP internalization, with G2/M > S > G0/G1.^21^ Moreover, Panet and colleagues showed that the cell volume is an additional factor to take into account since the cellular capacity for internalizing NPs seems to increase with size.^22^

In general, the process of NP internalization relies on the endocytic machinery of the cell. The standard routes followed by cells to internalize exogenous material are pinocytosis, phagocytosis, and receptor-mediated endocytosis.^10,23^ Pinocytosis is involved in the internalization of fluids or solutes, which includes many unrelated endocytic mechanisms, like clathrin-mediated, caveolar, nonclathrin/noncaveolar (clathrin-independent), lipid-raft-mediated, and micropinocytosis.^24^ Usually, NP uptake follows these well-known mechanisms and no NP-specific mechanisms have been identified to date.^10,25,26^

The mechanism of NPs internalization described in this study appears to follow a mechanism that is entirely distinct from phagocytosis and clathrin-mediated and clathrin-independent endocytosis. We investigated the internalization of PAA-functionalized ceria (ACNPs) and magnetite (iron oxide; IO) NPs and showed that they gained entry to the cell through a pathway mediated exclusively by microvillus-specific adhesion. Three different types of cells presenting microvilli have been investigated. To the best of our knowledge, this has not been described before. This mechanism is probably general enough to represent a potential alternative approach for controlled incorporation of NPs in a large number of cells.

## Results and Discussion

**Figure 1:**
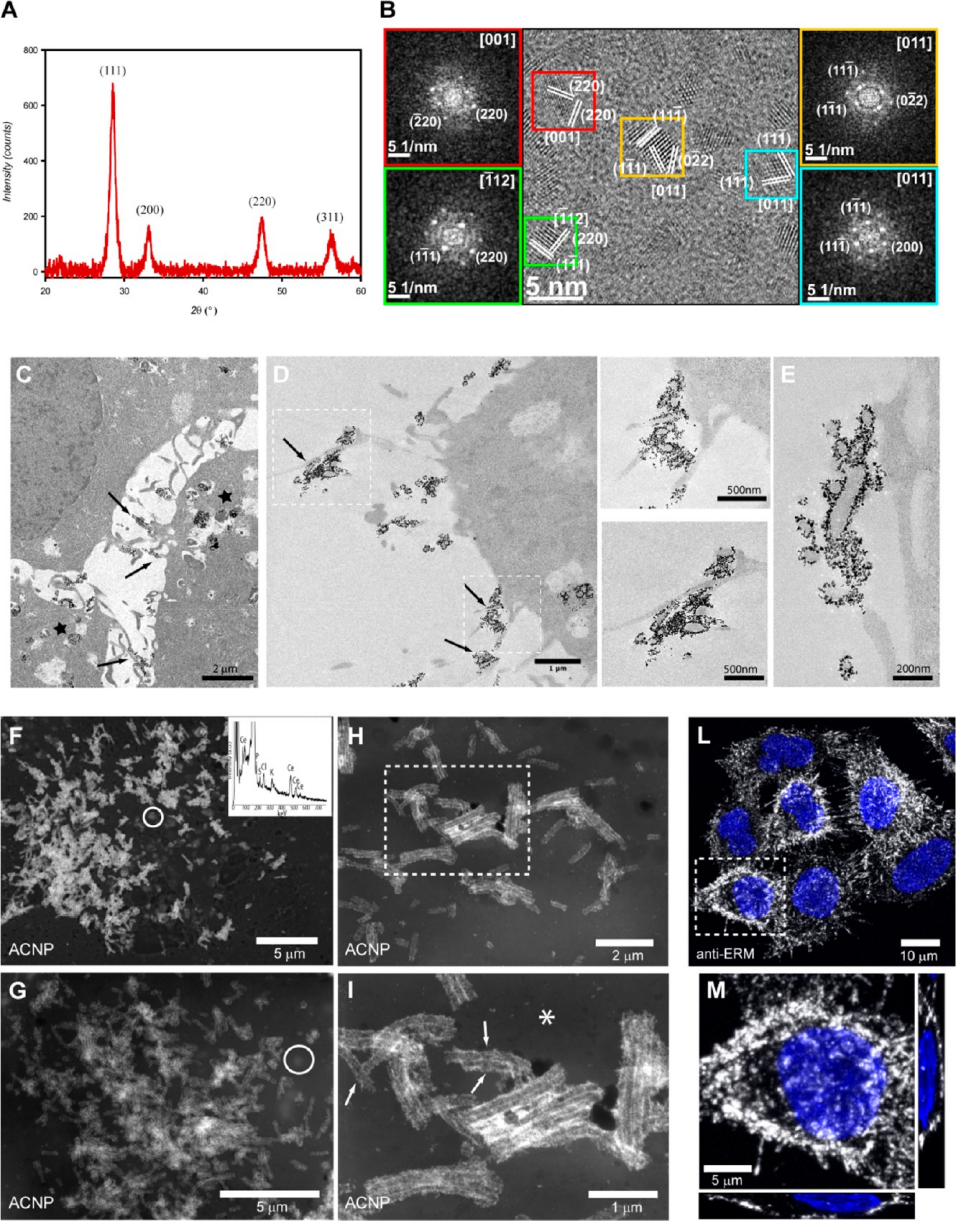
XRD analysis (A) and HRTEM imaging (B) of ACNPs. The insets with colored frames (B) show the individual NPs and their corresponding filtered 2D-FFT (numerical diffractograms). The crystal structure of the imaged NPs was ascribed to that of fluoritic CeO_2_. (C–E) TEM analysis of ACNP adhesion to HeLa cells. (C) HeLa cells incubated with ACNPs for 24 h. ACNPs, visible as black spots, were present on cell protrusions (arrows) and accumulated inside vesicles (stars). (D) On the cell surface, ACNPs decorated only the cell protrusions (arrows) without adhering to the planar region of the membrane. Microvilli covered with ACNPs, visible in (E), and two enlargements of (D), showed the specific interaction between ACNPs and microvilli. (F–I) HRSEM-BSE analysis of HeLa cells incubated with ACNPs for 24 h. (F inset) Representative SEM-EDS analysis of the ACNPs present on the cells. Signals other than Ce are due to the cell components and fixative. Due to the compositional contrast provided by the HRSEM-BSE imaging, ACNPs were revealed as bright spots on a darker background, making the ACNP-decorated cell protrusions visible (a single cell is shown in (F) and (G)). The use of a HRSEM accelerating voltage (8–10 kV) allowed visualization of only NPs on or just underneath the cell surface. However, only the superficial ACNPs could be imaged at the maximum resolution; ACNPs present in structures underneath the cell membrane looked blurred, as in the circled area in (F) and (G). Specificity of ACNPs toward the microvilli was better appreciated at higher magnification, (H, I) where ACNPs outlining the microvilli contours (arrows) became visible, while the rest of the membrane remained dark (*). (I) Enlargement of the area outlined in (H). (L, M) IF staining with pERM antibody (white) showed the presence of microvilli on the surface of HeLa cells. (M) Enlargement of area outlined in (L) and its relative cross sections, showing distribution of microvilli in a single cell. Blue, nuclei.

The ACNPs used in this study were obtained by direct precipitation from aqueous solution in the presence of an excess of PAA. The X-ray diffraction (XRD) pattern of the ACNPs (Figure 1A) showed the fluoritic CeO_2_ crystal structure (PDF 98-002-8709). High-resolution transmission electron microscopy (HRTEM) (Figure 1B), together with filtered bidimensional fast Fourier transform (2D-FFT) of several single particles, demonstrated that the ACNPs were monocrystalline and confirmed the fluoritic CeO_2_ crystal structure. HRTEM images showed neither evidence of internal defects nor of significant agglomeration and estimated the mean particle size to be 2.9 ± 0.9 nm (80%, 2–4 nm; 20%, 4–6 nm). The hydrodynamic diameter, as detected from dynamic light scattering (DLS) measurements, was significantly larger (14 ± 1 nm), accounting for the polymeric shell surrounding the ACNPs.

The peculiar interaction between these ACNPs and HeLa cells was clearly shown by TEM of Figure 1C–E. After 24 h incubation, the ACNPs were observed in two cellular regions: on the cell surface and in endolysosomal structures, confirming our previous observations.^27^

**Figure 2:**
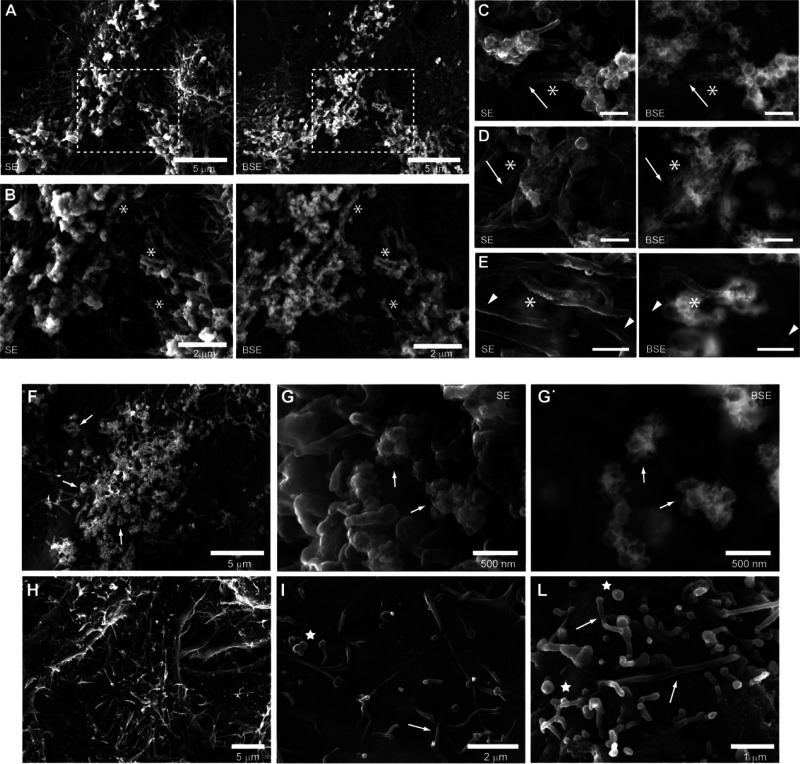
(A–E) HRSEM SE and BSE images of ACNP distribution in cells with preserved 3D morphology. The areas decorated by microvilli appeared brighter on both SE and BSE images. SE images showed the overall morphology better, while BSE showed only the areas where the ACNPs were present. (A) Cells treated with ACNP for 24 h. ACNP-decorated microvilli are mostly concentrated on top of the cell. (B) Enlargement of area outlined in (A). Microvilli were heavily decorated with ACNP (asterisk) but almost no ACNPs were visible on the underling planar membrane. (C, D) Examples of microvilli covered by ACNPs along their entire length (asterisk). The complete absence of ACNPs at the base of the decorated microvillus (arrows) showed high specificity for the microvillar membrane. Bar = 500 nm. (E) Detail of a microvillus decorated with ACNPs (asterisk) next to nondecorated microvilli (arrowheads). Bar = 500 nm. For each couple of images reported: left, SE image; right, BSE image. (F–L) ACNPs interacted with spherical membrane extrusions (buds), which together with microvilli, were not induced by ACNPs. (F) HeLa cell incubated with ACNPs for 24 h. ACNPs decorated the small bud-like structures (arrow) with high specificity, as for microvilli. (G, G′) Region rich in bud-like structures (arrow) covered with ACNPs. ACNPs interacted poorly with the surrounding planar membrane. (H, I) Control HeLa cells showed evident protrusions. Microvilli (arrow) and small bud-like structures (star) were less (I) or more (L) densely distributed. (G, H–L) SE image. (F, G′) BSE image.

However, the ACNPs on the cell membrane appeared specifically adherent to a few cell protrusions (Figure 1C,D). When observed at higher magnification (Figure 1D,E), the ACNPs appeared to form a uniform layer on the surface of these protrusions characterized by an internal diameter of about 75.33 ± 13.14 nm. Surprisingly, no evidence of adhesion on the planar portion of the plasma membrane was observed (Figure 1D,E). More complete characterization of ACNP distribution on the cell surface was achieved using high-resolution scanning electron microscopy (HRSEM). This approach avoided ultrathin sectioning of the entire cell, as required by TEM, to obtain whole-cell images of ACNP distribution. Alcohol-fixed samples were imaged by HRSEM, using both secondary electron (SE) and backscattered electron (BSE) detectors. The BSE imaging provided effective visualization of ACNP distribution, due to the significant difference in mean atomic number between the NPs and cellular structures (Figure 1F–I). This analysis confirmed that no ACNPs were present on the planar membrane of the whole cells, as ACNPs were exclusively attached to structures that resembled cell protrusions (Figure 1H,I). Shape, dimensions, and localization of these ACNP-decorated protrusions resembled those of cell microvilli.^28,29^ To confirm this, we analyzed by immunofluorescence (IF) the reactivity and distribution of the phosphorylated form of ezrin–radixin–myosin protein complex (pERM), which is known to be specific for microvilli and to regulate their formation.^30,31^ The detection of anti-pERM antibody on the surface of HeLa cells confirmed the nature of these cellular structures (Figure 1L,M). To further improve visualization of the ACNP–microvillus interaction, we also used a different type of fixation that preserved better the three-dimensional (3D) morphology of the cell surface that was otherwise not visible with alcohol fixation. This confirmed that the ACNPs (indicated by the brighter areas in Figure 2) adhered mostly to the microvilli present on the thicker part of the cell body and less to the processes in contact with the substrate (Figure 2A). Higher magnification showed that ACNPs covered the entire length of the microvilli (Figure 2B–E, asterisk) while leaving the rest of the cell membrane completely devoid of particles. A clear line of demarcation was visible at the base of the microvilli where ACNPs abruptly stopped adhering (Figure 2C,D, arrows).

The ACNPs had great affinity toward other small spherical protrusions (Figure 2C,F,G) with a diameter comparable to that of microvilli. It must be noted that these structures (buds), together with microvilli, were also present on control cells (Figure 2H–L), proving that they were not artifacts or formations induced by ACNPs. As for the microvilli, the specificity of ACNPs toward these structures was high (Figure 2G,G′). The similarity in the dimensions and affinity toward ACNPs leads us to speculate that the nature of this other type of protrusion is similar to that of microvilli. Similar spherical membrane extrusions have been reported to appear whenever cells are chemically treated to reduce formation of microvilli,^32^ suggesting that they represent buds of undeveloped or growing microvilli.^33^

Although all the ACNPs interacted with microvilli, not all microvilli interacted with the ACNPs. Some microvilli in close proximity to one another, where ACNP accessibility and concentration could be assumed to be similar, showed large differences in ACNP adhesion (Figure 2E, arrowhead). Such variability poses the question of why some of them were not “recognized” by the ACNPs. We speculate that microvilli in different stages (growing, stable, or shrinking) have different structure or membrane composition. In that case, ACNP adhesion represents selection for a specific membrane component.

To exclude the possibility that the NPs’ specificity toward microvilli was cell dependent, we investigated additional cell types other than HeLa, such as human epidermoid carcinoma cells (A431), human mesothelioma (MSTO and REN) cells, and monkey kidney-derived fibroblasts (COS-7). Affinity of the ACNPs toward the cell protrusions was evident in A431, REN, and MSTO cells, where the cell processes were heavily decorated (Supplementary Figure 1). No specific adhesion was observed in COS-7 cells that lacked microvilli on their surface. Here, ACNPs were distributed on the cell membrane as small patches.

The ACNP specificity was investigated using different concentrations (100, 200, and 500 μg/mL) (Supplementary Figure 2A) and different exposure times (5 min and 6 and 24 h) (Supplementary Figure 2B). The concentration of ACNPs, not toxic even for the longest incubation time (Supplementary Figure 2C), did not alter their specificity, and more interestingly, the specific adhesion was already evident after only 5 min exposure. This microvillus-mediated adhesion (MMA) was also analyzed at low temperatures when passive diffusion could not take place and the endocytic mechanisms were inhibited. ACNPs added to the cells preconditioned at 4 °C were still able to interact with microvilli during the subsequent 5 or 30 min incubation (Supplementary Figure 3). Although the specificity was still apparent, a less uniform layer of NPs was observed at 4 °C. The patchy distribution of the ACNPs could be explained by the redistribution of the lipid raft components to form larger clusters below 37 °C.^34^

To monitor the dynamics of ACNP adhesion to microvilli, we performed pulse–chase experiments that involved exposing the cells to ACNPs for 1 h (pulse) and then following distribution of the ACNPs for a further 2 h in a medium devoid of NPs (chase), as described previously.^27^ The life cycle of short microvilli of 500 nm, like those observed in HeLa cells, is ∼12 min.^33^ Therefore, at the end of the chase period no decorated microvilli should have been observed on the cell surface because they should have already been internalized or dismantled. In contrast, 2 h after removal of the ACNPs, decorated microvilli could still be detected (Supplementary Figure 4), suggesting that the dynamics of the ACNP-bearing microvilli could have been impaired or altered.

To clarify the nature of the interaction between ACNPs and microvilli, we designed a series of experiments that modified the characteristics of the NPs and incubation medium. First, we aimed to exclude the possibility that the interaction could be mediated by some constituent of the protein corona, generated on the surface of the ACNPs after exposure to the cell culture medium. It is well-known that the protein corona can alter or guide the interaction of NPs with biological structures.^35−38^ To reduce the exposure of our ACNPs to biological macromolecules, we performed the incubation in a balanced salt solution medium [Hanks’ balanced salt solution (HBSS) without fetal bovine serum (FBS)] instead of the regular cell culture medium [Dulbecco’s modified Eagle’s medium (DMEM) with FBS]. The change in culture medium produced only a slight modification in the hydrodynamic dimension of the ACNP (DLS: 8 ± 3 nm in DMEM; 13 ± 1 nm in HBSS. ζ potential: −20.6 ± 2 in DMM; −29 ± 0.2 in HBSS). With HBSS, we tested the ACNP interaction after 5 min and 6 and 24 h incubation (Supplementary Figure 5). Even under these conditions, decorated microvilli could still be detected, showing that protein corona was not fundamental for the adhesion of ACNPs. The amount of ACNPs with HBSS was even higher than with the regular culture medium. This could be explained by the increase in microvillus density as a consequence of starvation.^39^

**Figure 3:**
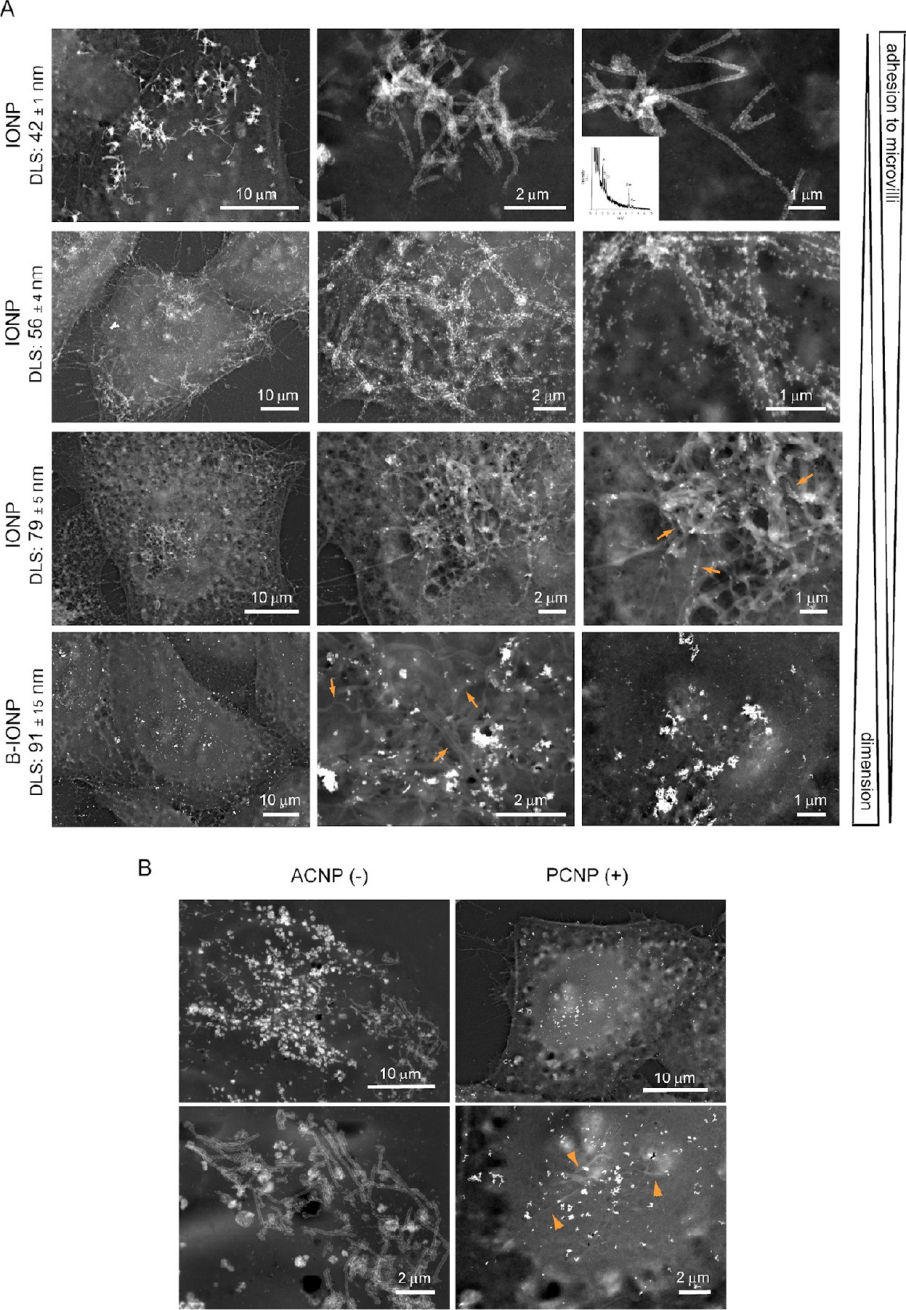
Influence of NPs surface charge, inorganic core chemical composition, and size on MMA. (A) The chemical nature of the NPs’ inorganic core did not affect MMA. NPs of PAA-coated Fe_3_O_4_ (IONPs) interacted with the microvillous surface in a way similar to ACNPs. (Inset) Representative SEM-EDS analysis of the IONPs present on the cells. MMA was instead affected by NP size. The affinity toward microvilli was reduced by increasing the size of IONP aggregates. The largest aggregates, including inert B_4_C NPs (B-IONP, lower row), did not show any interaction with microvilli. NP size was inversely proportional to the degree of MMA. (B) HRSEM-BSE images of cells treated with ACNPs and PCNPs. These NPs were negatively and positively charged, respectively. Different from ACNPs, PCNPs did not interact with microvilli (indicated by arrowheads).

We finally investigated whether MMA depended on the chemical nature of the inorganic core of the NPs and on their dimensions (Figure 3A). We produced NPs of PAA-coated Fe_3_O_4_ (IONPs, XRD 6–7 nm; DLS, 42 ± 1 nm). For XRD and HRTEM images see Supplementary Figure 6. These NPs showed an affinity toward microvilli similar to that presented by the ACNPs, covering their surface with a uniform layer (Figure 3A). To evaluate the role of the NPs’ dimensions, we used the same IONPs but with different levels of aggregation. The larger aggregates (DLS, 56 ± 4 nm) still decorated the microvilli but formed a discontinuous layer (Figure 3A). The discontinuous adhesion indicated a lower ability to interact with the membranes of the microvilli. With the IONPs largest aggregates (DLS, 79 ± 5 nm), the specificity was further reduced. However, when the size was increased further, using composite NPs presenting a boron carbide core (B) covered with IONPs (B-IONPs) (DLS, 91 ± 15 nm), we did not detect any specific adhesion on the cell protrusions (Figure 3A). In all three cases, the NPs presented the same constitutive elements represented by small IONPs, showing by themselves a strong affinity toward microvilli; therefore, it can be concluded that the observed different behavior was due to the NPs’ dimensions. The size cutoff for MMA can probably be placed between 70 and 80 nm.

To verify the role of PAA in defining the selective interaction of ACNPs with microvilli, we investigated the adhesion of CNPs with different kinds of functionalization. By substituting PAA with dextran, we produced NPs with similar size but ionically neutral surface (dextran-coated CNPs, DCNPs; XRD, 3 nm; DLS, 6.5 ± 0.5 nm). When these DCNPs were added to the cells, we did not observe any specific adherence to microvilli (Supplementary Figure 7). Excluding altogether PAA from ACNP synthesis, we obtained similar results, with NPs that were randomly distributed on the cell surface as a few large aggregates (Supplementary Figure 7). Loss of specificity toward microvilli was also observed when positively charged CNPs (PCNPs, XRD, 1.8 nm; DLS, 39 ± 2 nm; ζ potential, +20 ± 0.6) were used (Figure 3B). Even in this case, PCNPs were observed on the cell surface without any apparent specific distribution.

The absence of MMA using all these NPs is a clear indication that the negative, polyanionic characteristic of PAA is an essential element in controlling.

It is difficult to pinpoint precisely what makes PAA the mediator of such a specific interaction and to identify the components of the microvilli partnering with PAA. Microvilli are characterized by specific components that differ from the rest of the cell planar membrane.^31,32,40−44^ The microvillus membrane is characterized by different organization of lipid rafts and some associated proteins,^32,42^ and the phospholipid distribution in the outer and inner leaflets of the microvillus membrane differs from that of the rest of the membrane.^44^ Formation of microvilli requires the low-curvature cell membrane to be transformed into a high-curvature membrane. Such change induces a shift of phospholipids in the inner leaflet of the membrane to the outer leaflet to fulfill the spatial requirements, with the transition zone being at the base of the growing microvilli.^44^

**Figure 4:**
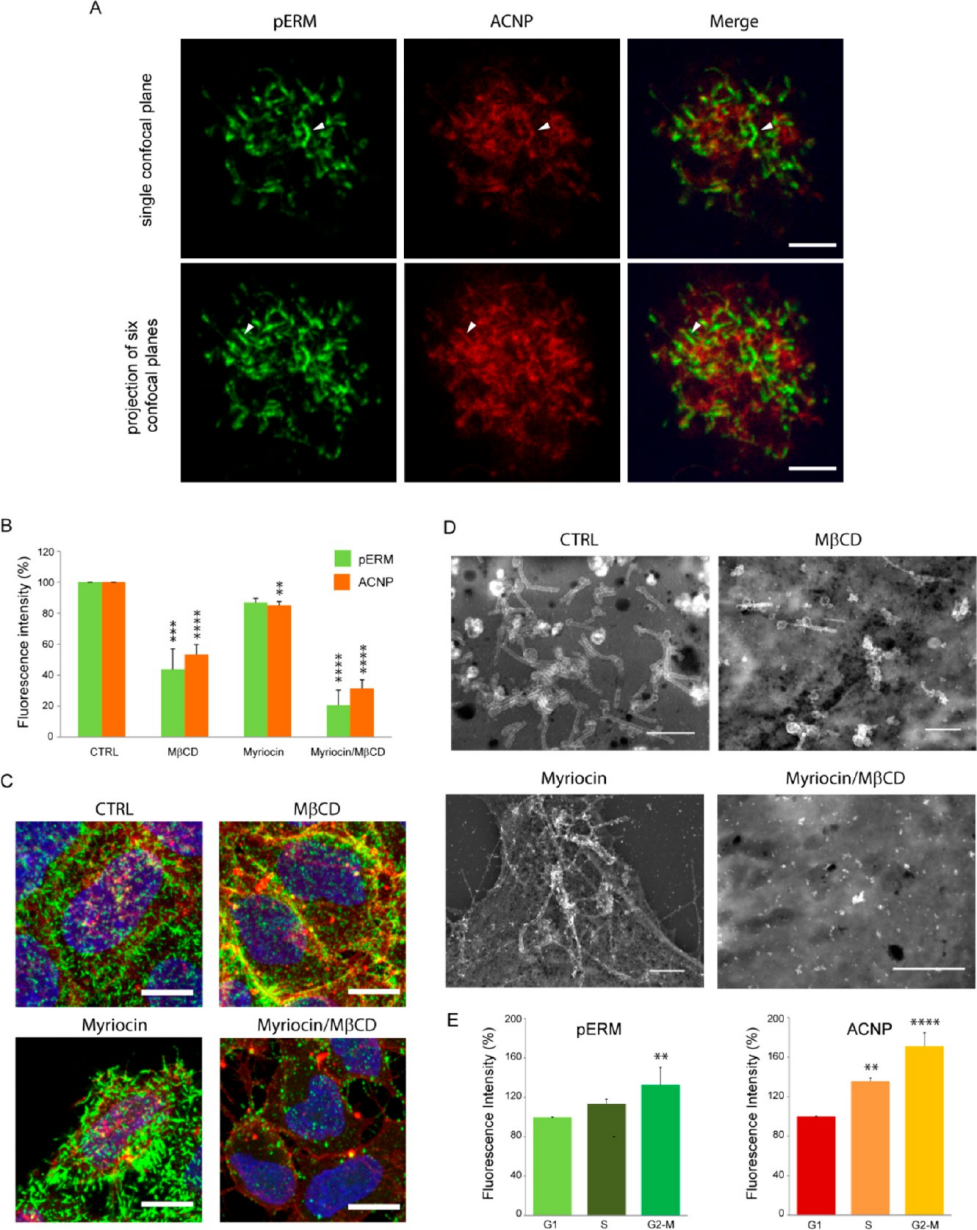
Microvilli and lipid rafts are fundamental for NP–cell interaction. (A) ACNPs and pERM reciprocal distribution shown by IF. ACNPs (red) were present on the cell surface in a spaghetti-like distribution, as for pERM (green). Areas of colocalization with pERM, a component of microvilli membranes, are visible (yellow) in the Merge images (some indicated by arrowheads). Single confocal plane, 0.35 μm. Bar = 5 μm. (B–D) Effect of lipid raft alteration on ACNP adhesion to microvilli. (B) Flow cytometry measurement from three independent experiments of pERM and ACNP levels in cells with reduced cholesterol level (MβCD), phospholipids (myriocin), or both (myriocin/MβCD). ∗∗, *p* < 0.01; ∗∗∗, *p* < 0.001; ∗∗∗∗, *p* < 0.0001 versus the respective control. (C) Confocal images of cells treated as in (B). Maximum projection of 15–18 0.35 μm confocal slices. Green, pERM; red, ACNP; blue, nuclei. Bar = 10 μm. (D) HRSEM BSE images of cells treated as in (B). Bar = 2 μm. (E) The amount of ACNP adherence to the cell surface changed with cell cycle (G2/M > S > G1) and correlated with pERM. Measurement from three independent experiments. ∗∗, *p* < 0.01; ∗∗∗∗, *p* < 0.0001 versus the respective G1 column.

Besides the phospholipid distribution and, more specifically, the sphingomyelin clustering, which is essential for microvillus formation,^45^ other specific proteins like pERM define the microvillus domain.^30,31^ In addition to cross-linking actin filaments with the plasma membrane, pERM is a constituent of the lipid rafts that plays an essential role in microvillus maintenance.^32^ To identify the components of the microvillus membrane that are involved in ACNP adhesion, ACNPs were made fluorescent by adding the lipophilic dye 1,1′-dioctadecyl-3,3,3′,3′-tetramethylindocarbocyanine perchlorate (DiI) in the PAA capping,^9^ and their distribution was investigated with respect to pERM. Short incubation times of 15 min were used to focus exclusively on the adhesion process, and the ACNPs showed a distribution similar to that of pERM (Figure 4A). These results support a possible interaction between PAA, on the ACNP surface, and one of the components of lipid rafts, which are differently organized and more abundant in microvilli than in planar membranes. Density of microvilli is affected by treatment that alters the membrane cholesterol composition, as lipid rafts are involved in the formation of microvilli.^32^ To confirm this, we removed phospholipids and/or cholesterol and evaluated the resulting pERM and ACNP levels by flow cytometry. Cholesterol depletion by methyl-β-cyclodextrin (MβCD), alone or in combination with a reduced level of sphingolipid induced by myriocin, decreased the pERM signal and ACNP level (Figure 4B), confirming their role in defining the affinity toward ACNPs. However, modification of sphingolipid synthesis alone did not induce any reduction in pERM level and only slightly reduced ACNP adhesion. IF and HRSEM were used to visualize how these modifications in membrane composition correlated with pERM and ACNP distribution (Figure 4C,D). The effectiveness of MβCD or myriocin/MβCD was confirmed by IF. pERM was almost completely absent from the cell surface and the amount of ACNPs was greatly reduced, or they were only visible as small concentrated spots. HRSEM confirmed that microvilli were almost completely eliminated. The only ACNPs left were present on spherical structures or remnants of microvilli, accounting for the ACNP aggregates visible by IF. In the case of membranes depleted only of sphingolipids, IF confirmed the presence of dense microvilli with adherent ACNPs, which by SEM showed a less dense distribution. This observation confirms that although the alteration of phospholipid alone did not reduce formation of microvilli, it did alter ACNP adhesion, showing clearly the importance of lipid rafts for MMA. The reduction in ACNP adhesion was not due to a reduction in microvillus density (as for MβCD or myriocin/MβCD treatments) but to the change in their membrane composition.

We tried also to clarify if ACNP adhesion was dependent on the status of the cell cycle. Microvillus density on HeLa cells correlates with the cell cycle.^28^ We evaluated by flow cytometry the ACNP level or pERM signal in relation to DNA content. After 15 min incubation, ACNP adhesion was higher in G2/M than S phase, which in turn was higher than in G1 phase, as it was for the pERM signal (Figure 4E).

**Figure 5:**
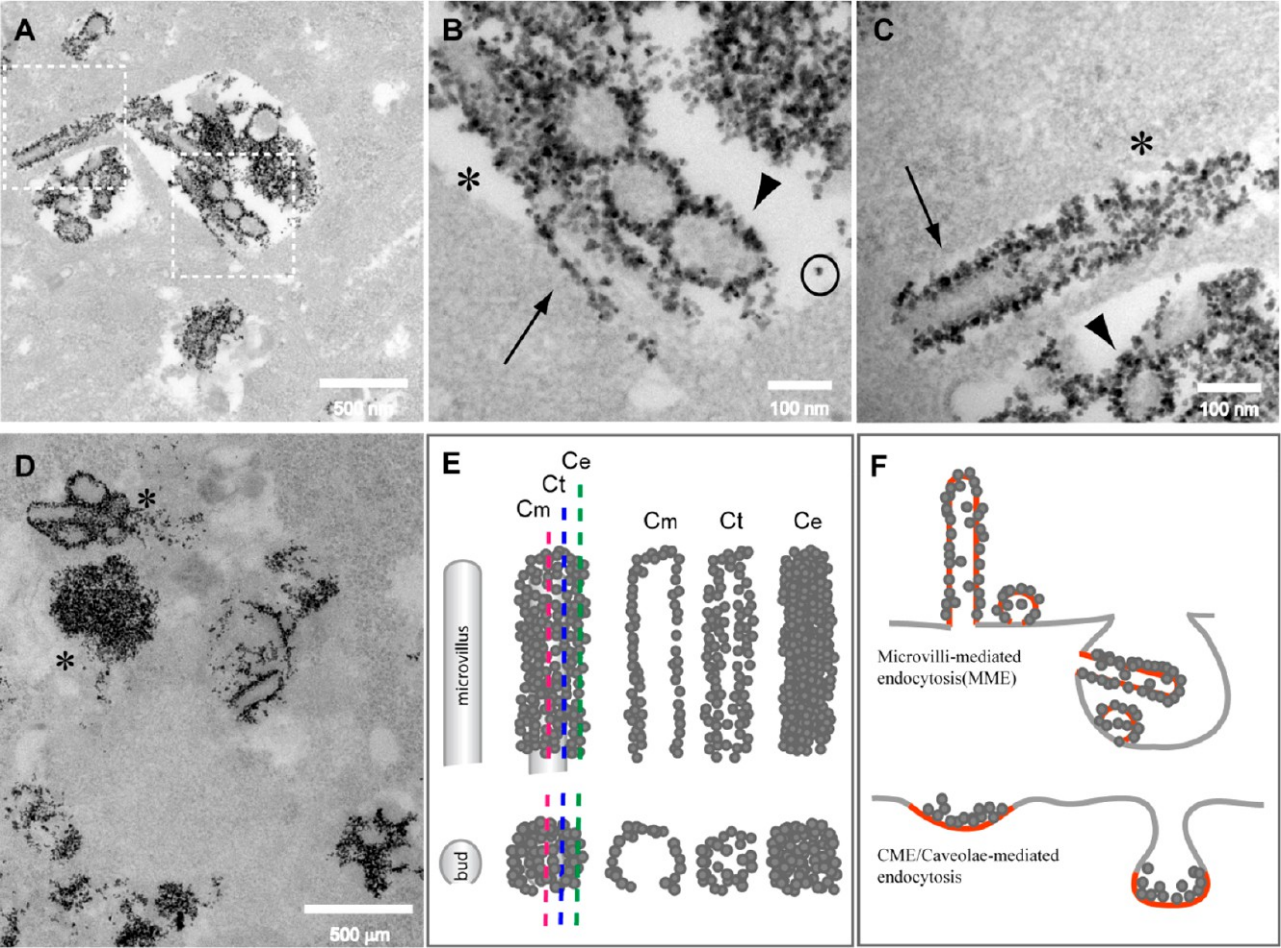
TEM analysis of ACNP internalization. (A) After 24 h incubation, the internalized ACNPs were concentrated inside endolysosomal structures. (B, C) Enlargement of areas outlined in (A) shows only a few free ACNPs inside the vesicles (circle), with most being adherent to cylindrical structures resembling microvilli (arrows) or bud-like structures (arrowhead) that mediated their extracellular adhesion (B, C). (D) Larger ACNP aggregates derived from several microvilli/buds assembled together (∗). (E) ACNP-covered microvilli/buds appeared different depending on the position of the sectioning plane. When sectioned in proximity to the external surface, only a uniform layer of NPs was observed (Ce and regions marked by ∗ in (B) and (C)). The internal structures of microvilli or buds began to appear if the cutting crosses tangentially (Ct) the microvilli surface. When the section crossed in the middle of the microvilli or buds (Cm), the ACNPs appeared as a granulated uniform layer outlining their perimeter (arrow and arrowhead in (B) and (C)). (F) Schematic representation of the difference in NP internalization between classical endocytosis and internalization following MMA. In MMA, the membrane where NPs adhered (red) did not become part of the membrane of the newly formed endocytic vesicle. In contrast, in internalization *via* CME/caveolae endocytosis, the membrane that promotes NP adhesion (red) participated in forming the vesicle membrane.

ACNP internalization by MMA presents also some unusual characteristics. Following adhesion to microvilli, ACNPs are transferred into endolysosomal structures, whose number and dimensions increase with time.^27^ Surprisingly, within these structures, the ACNPs appear to be still mostly attached to portions of the microvilli or buds of undeveloped or growing microvilli (Figure 5). The diameter of the ACNP-decorated structures observed in the endolysosomes was comparable to that of the microvilli/buds observed on the cell surface (75.88 ± 14.17 vs 74.60 ± 13.25 nm, respectively). The diameter of the microvilli and buds was determined only on structures similar to those shown in Figure 5C (arrow, microvilli; arrowhead, buds) whose structure is schematized in Figure 5E as Cm. Decorated structures indicated by an asterisk in Figure 5C and schematized in Figure 5F as Ct and Ce were not considered for the measurements. This observation suggests that once “decorated” by ACNPs, microvilli and buds are not disassembled but are internalized as a whole, probably being perceived as material to be eliminated. This type of internalization is different from that generally reported, in which NPs are captured as random agglomerates or attached to the inner side of the vesicular membrane.^8,26,46^ This provides direct evidence that adhesion to microvilli represents an alternative internalization process.

Altogether, our results support a PAA-mediated mechanism for the internalization of small NPs that depends on the presence and specific characteristics of microvilli and might be modulated by the cell cycle. MMA seems to represent an alternative route/process that shares some of the characteristics of phagocytosis, pinocytosis, and clathrin/caveolin-mediated endocytosis. In analogy with the mechanisms involving phagocytosis and macropinocytosis, MMA favors NP entry to the cell, while, like clathrin/caveolin-mediated endocytosis, it relies on specific recognition represented, in this case, by the microvillus membrane. However, the microvillus membrane does not become part of the newly formed vesicle/macropinosome membrane, as it does for phagocytosis, pinocytosis, and clathrin/caveolin-mediated endocytosis (Figure 5F). The decorated microvilli appear to be ingested by the cell as a whole and do not provide any structure to the vesicle. It can be speculated that, in a type of defensive mechanism, the cell actively internalizes these NP-carrying microvilli in an attempt to eliminate the exogenous material that is attached to them. Additionally, unlike the phagocytotic and macropinocytotic mechanisms, microvilli are not formed in response to an NP-driven stimulus. Although we cannot rule out that, for longer incubation times, the number of microvilli can increase in response to the presence of ACNPs, it appears that, for short incubation times, microvilli are not specifically formed to favor NP uptake.

## Conclusion

MMA can be considered an alternative pathway for NP internalization that does not depend on the chemical nature of the NPs or the cell types. The specificity of the process is, however, influenced by the size and surface functionalization of the NPs. The interaction mechanism of polyacrylic acid-functionalized NPs with microvilli depends on a specific component of microvilli membrane, possibly involving the interaction of microvillus lipid rafts with the nanoparticle functionalization. MMA might represent an interesting alternative route for drug delivery in the case of epithelial cells that abound in microvilli, such as gastric and intestinal epithelia. In cells such as HeLa, in which the density and distribution of microvilli correlate with the cell cycle, this specific adhesion could be exploited to obtain delivery controlled by the status of the cell.

## Methods

### Nanoparticle Synthesis and Characterization

The ACNPs were produced by direct precipitation from aqueous solution and stabilized in the presence of PAA, as described previously.^27^ Ce(NO_3_)_3_·6H_2_O was dissolved in distilled water, and 2% PAA solution was added to obtain a 2:1 (v/v) ratio. Concentrated NH_4_OH solution was added dropwise to obtain pH ∼12, and the solution was kept under constant stirring for 48 h. The final ACNP suspension had a CeO_2_ content of ∼6 mg/mL, as determined by inductively coupled plasma optical emission spectrometry (ICP-OES) analysis. To remove excess PAA, the suspension was centrifuged at 13 000*g* for 30 min and the pellet recovered in deionized water.

The IONPs were produced by direct precipitation of Fe_3_O_4_ from aqueous solution in the presence of PAA, as previously described.^47^ We prepared an iron salts solution by dissolving 0.18 g of FeCl_2_·4H_2_O and 0.37 g of FeCl_3_·6H_2_O in 2 mL of 0.5 N HCl. This was added to 15 mL of distilled H_2_O, and under vigorous stirring we dropped in 2 mL of NH_4_OH (30% NH_3_). The resulting dark suspension of IONPs was stirred for 30 s before adding 5 mL of PAA solution (9.4% w/v) and stirred for 1 h again. The final suspension of PAA-IONPs was centrifuged at 1000*g* for 30 min to remove the largest aggregates. The supernatant was centrifuged at 25 000*g* for 30 min to remove the free PAA and other unreacted reagents. The pellet was washed twice and, eventually, recovered in deionized water. To obtain IONPs with different levels of aggregation, IONPs were sequentially centrifugated at 5000*g* and 10 000*g* for 30 min. Each fraction was recovered in deionized water and characterized by DLS.

The B-IONPs were obtained by modifying the procedure used for IONPs and precipitating Fe_3_O_4_ in the presence of B_4_C NPs. Commercial B_4_C nanopowder (SkySpring Nanomaterials Inc., Houston, TX, USA) was subjected to a ball milling treatment using a planetary mill (Fritsch Pulverisette 7 premium line) with tungsten carbide jar and balls, to reduce agglomeration. The obtained nanopowder was recovered in deionized water. This suspension of B_4_C (3 mg/mL) was added to the stirring iron salts solution prepared as above, before NH_4_OH and PAA incorporation. After 1 h, the resulting suspension was centrifuged at 300*g* for 30 min to remove the largest aggregates and the supernatant was centrifuged at 15 000*g* for 30 min to remove unreacted reagents. The pellet was washed twice and, eventually, recovered in deionized water.

The DCNPs were produced similarly to ACNPs except for the substitution of PAA with 10 mL of dextran solution (16.21% w/v; dextran from *Streptococcus mutans*, average mol wt 9000–11 000; Sigma-Aldrich, St. Louis, MO, USA).

PCNPs were synthesized by direct precipitation from aqueous solution and functionalized with polyethyleneimine (PEI), as reported by Turin-Moleavin et al.^48^ Briefly, 2.50 mL of 0.1 M solution of Ce(NO_3_)_3_·6H_2_O was added dropwise under magnetic stirring to a mixture of 1.25 mL of 0.1 M solution of PEI (800 Da-branched) with 7.50 mL of NH_4_OH (28–30%). The mixture was left under continuous magnetic stirring for 24 h. After completion of the reaction, the suspension was centrifuged at 17 000*g* for 10 min and washed with deionized water. The supernatant was centrifuged at 25 500*g* for 15 min, and the pellet was washed three times with distilled H_2_O.

For the preparation of fluorescent ACNPs, DiI fluorescent dye (Sigma-Aldrich, St. Louis, MO, USA) was dissolved in dimethylsulfoxide (1.2 mg/mL) and then added under stirring to the 6 mg/mL ACNP suspension (1:20 v/v), as described previously.^9^ The ACNP suspension was centrifuged at 17 000*g* for 20 min to remove free DiI in solution, and the pellet was recovered in deionized water.

ACNP, IONP, B-IONP characterization included XRD, DLS, ζ potential, and HRTEM analysis.

The CeO_2_ content was determined by ICP-OES analysis (ICP-OES Optima 3300 D; PerkinElmer, Santa Clara, CA, USA).

XRD analysis was performed on films obtained by evaporating 150 μL of ACNP, IONP, B-IONP suspensions on glass microscope slides. The XRD patterns were acquired using a Bruker D8 Advance diffractometer (Bruker Corp., Billerica, MA, USA) with a Cu anticathode (λ(Cu Kα) = 1.541 838 Å) operated at 40 kV and 40 mA. Diffractograms were acquired in θ–θ mode, with a step of 0.03° 2θ and an acquisition time of 20 s per step.

The hydrodynamic diameter and ζ potential of the NPs were evaluated with a Nano ZS90 DLS apparatus (Malvern Instruments, Malvern, U.K.). For DLS, three measurements were performed on diluted solutions (∼1 mg/mL) for each sample, providing average sizes, distribution widths, polydispersion index, and associated standard deviations (SDs). For ζ potential, Zetasizer disposable folded capillary cells were used (Malvern Instruments).

For HRTEM, a drop of NP suspension was placed on an ultrathin, carbon-membrane, 400-mesh copper grid and left to dry for 5 min. HRTEM imaging was performed using an FEI Titan 80-300 Cube transmission electron microscope (Hillsboro, OR, USA), operating at an accelerating voltage of 300 kV, equipped with S-Twin objective lens, an FEI X-FEG Schottky electron source, a 2K × 2K US1000 Gatan CCD camera, and an EDS Si(Li) EDAX detector.

### Cells and Treatment

HeLa cervical cancer cells, MSTO and REN human mesothelioma cells, A431 human epidermoid carcinoma cells, and COS-7 monkey kidney-derived fibroblasts were maintained in DMEM with 10% FBS and 2 mM l-glutamine (Lonza, Basel, Switzerland) at 37 °C in a humidified atmosphere of 5% CO_2_ in air.

When treated with NPs, cells were seeded at 3.0 × 10^5^ in 9 cm^2^ Petri dishes. When 50% confluency was reached, cells were washed and incubated with fresh medium containing ACNP for the time specified, at a concentration of 200 μg/mL or as otherwise indicated.

For pulse–chase experiments, HeLa cells were incubated with ACNP for the indicated time (pulse), and after removal of ACNP and extensive washing, cells were maintained in culture for an additional 2 h (chase). At the end of the chase period, cells were processed for SEM.

For treatment at low temperature, HeLa cells were preconditioned for 30 min at 4 °C. After this period the ACNPs were added to the cold culture medium and cells were incubated at 4 °C for 5 or 30 min.

### MTT Cytotoxicity Assay

For testing cell viability after CNPs treatment, MTT (3-(4,5-dimethylthiazol-2-yl)-2,5-diphenyltetrazolium bromide; Sigma-Aldrich, St Louis, MO, USA; 0.9 mM) was added to the cells and incubated for 2 h at 37 °C. The medium was then removed and 100 μL of DMSO was added to dissolve the blue formazan that was formed. Cell viability was assessed by measuring absorbance at 540 nm (Bio-Rad microplate reader, Hercules, CA, USA) and expressed as a percentage of the control group (set to 100%). The data were presented as the mean of three independent experiments.

### Alteration of Microvillus Membrane Composition

For sphingolipid and cholesterol content alteration, myriocin and MβCD were used as previously described.^32^ To extract cholesterol, HeLa cells were incubated with freshly prepared 10 mM MβCD (Sigma-Aldrich) in serum-free medium for 30 min. To inhibit sphingolipid synthesis, cells were grown with 10 μM myriocin (Sigma-Aldrich) for 48 h. For modification of both cholesterol and glycosphingolipid, cells were treated with myriocin first and then the myriocin-containing medium was substituted with serum-free medium containing 10 mM MβCD. For all treatments, at the end of incubation, MβCD- or myriocin-containing medium was removed and complete medium added with or without ACNP.

### IF and Light Microscopy

Cells grown on 18 mm × 18 mm glass coverslips and incubated with fluorescent ACNPs were processed for IF, as previously described.^27,49^ Cells washed with phosphate buffered saline (PBS) were fixed in 4% formaldehyde for 15 min. After permeabilization with 0.2% saponin for 10 min, samples were incubated for 30 min with rabbit anti-pERM primary antibody (1:200; Cell Signaling Technology, Leiden, The Netherlands) and then with fluorescent secondary antibody Alexa488-labeled anti-rabbit (1:400; Jackson Immunoresearch, West Grove, PA, USA). Nuclei were counterstained with Hoechst 33342 (Sigma-Aldrich). Samples were analyzed with a TCS SP8 confocal laser scanning microscope equipped with an HC PL APO CS2 40×/1.30 oil-immersion objective (Leica Microsystems, Heidelberg, Germany). Images were processed using ImageJ software and related plugins (National Institutes of Health, Bethesda, MD, USA).

### Flow Cytometry

HeLa cells were seeded in a 25 cm^2^ culture flask at 6.0 × 10^4^ cells/cm^2^. After 24 h cells were treated with or without ACNPs and then detached using trypsin–EDTA and recovered in sterile tubes. After fixation for 15 min in 10% formalin in PBS without Ca and Mg and permeabilization in 0.2% saponin, they were processed for antibody staining and flow cytometry.

We used an Attune NxT acoustic flow cytometer equipped with violet (405 nm, 50 mW) and blue (488 nm, 50 mW) lasers (Invitrogen, Carlsbad, CA, USA) and a BD FACSLyric flow cytometer equipped with violet (405 nm, 40 mW), blue (488 nm, 20 mW), and red (640 nm, 40 mW) lasers (BD Biosciences, Franklin Lakes, NJ, USA). For each sample, at least 3.0 × 10^5^ events were acquired at a flow rate of 200 μL/mL. Emission from the ACNPs was stimulated with the blue laser, and the fluorescence was detected using the band-pass filter (Attune NxT Acoustic, 574/26 nm; BD FACSLyric, 586/42 nm). For pERM, stained with Alexa488-labeled anti-rabbit secondary antibody (Jackson Immunoresearch, West Grove, PA, USA), we used blue laser excitation and the bandpass filter (530/30 nm) for detection with Attune NxT; for pERM stained with Alexa647-labeled anti-rabbit secondary antibody (Jackson Immunoresearch, West Grove, PA, USA) and analyzed by BD FACSLyric we used red laser excitation and the band-pass filter (660/10 nm) for detection. To measure the total DNA content, FxCycle Violet (Thermo Fisher, USA) was used. It was excited with the 405 nm laser, and the signal was detected in the violet channel (bandpass filter of 440/50 nm). Single-stain samples were prepared for compensation. Experiments have been carried out in a three biological replicates. The data presented are the results of three independent experiments.

### TEM

Cells were grown on 35 mm Petri dishes and directly fixed on the substrate by 2% glutaraldehyde in 0.1 M cacodylate buffer (pH 7.3) for 20 min at room temperature. The fixative was removed, and cells were extensively washed with 0.1 M cacodylate buffer. Cells were exposed to 1% aqueous OsO_4_ for 20 min at room temperature for secondary fixation and washed in pure distilled water. Cell monolayers were exposed to 1% uranyl acetate aqueous solution for 20 min at room temperature, washed with pure distilled water, and dehydrated through a graded series of ethanol (25, 50, 70, 90, 95, and 100%; 5 min each). Infiltration was carried out by placing cell monolayers in Durcupan ACM resin with ethanol as solvent and then progressively increasing the resin concentration, that is, 25% resin + 75% ethanol, 50% resin + 50% ethanol, and 75% resin + 25% ethanol, for 90 min each, and 100% resin, overnight at room temperature. Monolayers were finally embedded with fresh pure resin and after 2 h at room temperature moved to 60 °C for 48 h. Ultrathin sections (70 nm) were cut with a Leica EM UC7 ultramicrotome (Wetzlar, Germany), placed on 300-mesh copper grids, stained with lead citrate, and washed with pure distilled water. Samples were imaged by a FEI Tecnai Spirit electron microscope, operating at an accelerating voltage of 120 kV, equipped with a Bio-Twin objective lens, a thermionic LaB_6_ electron source, a 4K × 4K FEI Eagle CCD camera, and an EDS Si(Li) EDAX detector.

### HRSEM

Two different fixation procedures have been used for HRSEM analysis. For ethanol fixation, the adherent cells were extensively washed with PBS and fixed with cold 70% ethanol at −20 °C for at least 2 h. For observation, ethanol was removed and the cells were dried in air before being processed for SEM. To preserve the 3D architecture, cells were fixed with hexamethyldisilazane (HMDS; Sigma-Aldrich). Adherent cells were extensively washed with PBS and then with 0.05 M cacodylate buffer, pH 7.3. Fixation was performed for 2 h in 2.5% glutaraldehyde in cacodylate buffer at room temperature. Cells were dehydrated by increasing concentration of ethanol (from 70 to 100%) for 15 min each. Drying was performed with HMDS, in substitution of the critical point method.^50^ After two 15 min incubations in 100% ethanol, ethanol was substituted with a 1:2 solution of HMDS/100% ethanol and replaced with a 2:1 solution of HMDS/100% ethanol and finally 100% HMDS. The duration of these incubations was 20 min. Almost all the HMDS was removed, and only a thin layer of silane was left covering the bottom of the Petri dish that was kept open for 18–24 h to allow complete evaporation of HMDS. All steps including the use of glutaraldehyde or HMDS were performed in a fume hood.

All samples were carbon-coated and observed with a field-emission gun, high-resolution scanning electron microscope (Mira3 XMU; Tescan, Kohoutovice, Czech Republic) equipped with an EDAX EDS microprobe and SE and BSE detectors. The SE signal provided imaging of the cell surface morphology (morphological contrast). The BSE signal, being sensitive to local mean atomic number, was able to image the NPs (compositional contrast). The microscope operated at different voltages, depending on the information needed. To visualize the NPs present only on the cell surface, an accelerating voltage of 8–10 kV was used, while 15 kV was used to visualize also NPs inside the cells.

### Statistical Analysis

The flow-cytometry analysis was performed using FlowJo software (FlowJo software version X.0.7; Ashland, OR; Becton, Dickinson and Company; 2019). First, cell population was gated in the SSC-FSC panel, and singlets were then selected for subsequent analysis by gating in the FSC-A vs FSC-H panel. Distributions for respectively the FCNP and the pERM compensated signals were extracted for control and treated samples.

Parameters related to the cell cycle phases were extracted by means of subgating the cell cycle profile from control (“Ctrl”) singlet cells to define the three phases G1/G0, S, and G2/M. The FCNP and pERM signals were then extracted for each cell cycle phase.

All extracted distributions were highly asymmetric; thus the median was chosen as indicator of centrality. The data analysis was performed with framework ROOT.^51^ The medians were normalized to “Ctrl” values and to the value in the “G1/G0” phase, for Figure 4B and Figure 4E, respectively. Resulting values from the three different experiments were averaged, and the standard deviation was calculated. In Figure 4B, statistical significance was determined using one-way ANOVA for each signal independently, with Dunnets’s multiple comparisons test compared to the “Ctrl” sample. In Figure 4E, statistical significance was determined using one-way ANOVA for each signal independently, with Tukey’s multiple comparisons test.

## Supporting Information Available

ACNP adhesion to different cell types (Supplementary Figure 1); ACNPs adhesion for different concentrations and incubation times (Supplementary Figure 2); effect of low temperature on ACNP adhesion (Supplementary Figure 3); persistence of ACNPs on the cell surface in pulse-chase experiments (Supplementary Figure 4); ACNP adhesion not mediated by components present in the culture medium (Supplementary Figure 5); XRD analysis and HRTEM imaging of IONPs (Supplementary Figure 6); dextran coating reduced NP–cell interaction (Supplementary Figure 7) (PDF)
