# Supplementary material for: Microvilli Adhesion: An Alternative Route for Nanoparticle Cell Internalization
Source: ACS Nano. 2021 Sep 29;15(10):15803–14. doi: 10.1021/acsnano.1c03151 (PMC8552441; doi:10.1021/acsnano.1c03151)
Supplement: Supplementary file 1 — nn1c03151_si_001.pdf [file nn1c03151_si_001.pdf]

Supplemental Materials

Microvilli Adhesion: An Alternative Route for Nanoparticle Cell Internalization

Patrizia Sommi[1#*], Agostina Vitali[#2], Stefania Coniglio[1], Daniele Callegari[2], Sofia Barbieri[3], Alberto Casu[4], Andrea Falqui[4], Lorenzo Vigano'[2], Barbara Vigani[5], Franca Ferrari[5], Umberto Anselmi-Tamburini[2]

[1] Department of Molecular Medicine, Human Physiology Unit, University of Pavia, 27100 Pavia, Italy.
[2] Department of Chemistry, University of Pavia, 27100 Pavia, Italy
[3] Department of Physics, University of Pavia, 27100 Pavia, Italy
[4] King Abdullah University of Science and Technology (KAUST), Biological and Environmental Sciences and Engineering Division, NABLA Lab, 23955-6900 Thuwal, Saudi Arabia
[5] Department of Drug Sciences, University of Pavia, 27100 Pavia, Italy

[#]Shared first authorship

*Corresponding author: Patrizia Sommi PhD, Department of Molecular Medicine, Human Physiology Unit, University of Pavia, via Forlanini 6, Pavia 27100, Italy. Tel: +39 0382 987665
E-mail:patrizia.sommi@unipv.it

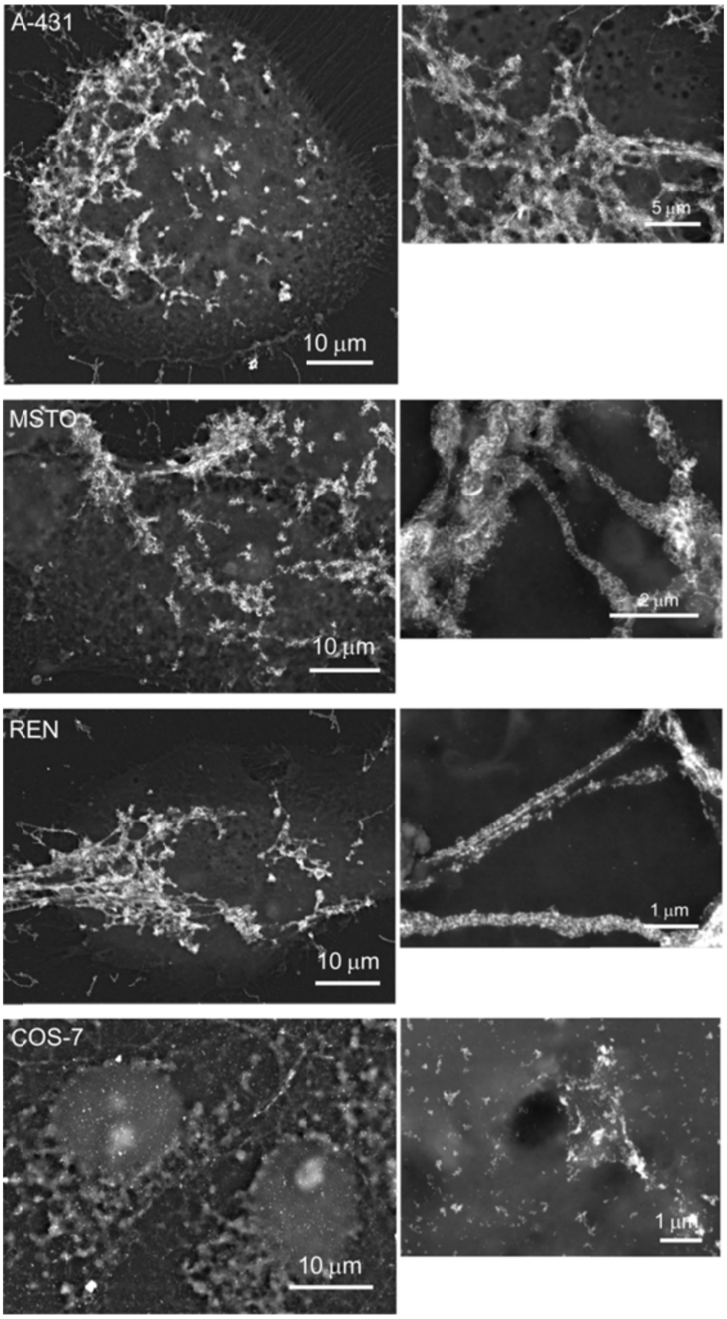

Supplementary Figure 1. ACNP adhesion to different cell types. Similarly for HeLa cells, ACNPs (100 µg/mL, 10 min) showed specific adhesion to other cell lines (A-431, MSTO and REN). As for HeLa cells, the ACNPs adhered specifically to cell processes without interacting with the planar membrane. No specific adhesion was observed in the case of COS-7 cell line, where ACNPs were distributed on the entire cell bodies without showing any specificity for particular structures. HRSEM-BSE images.

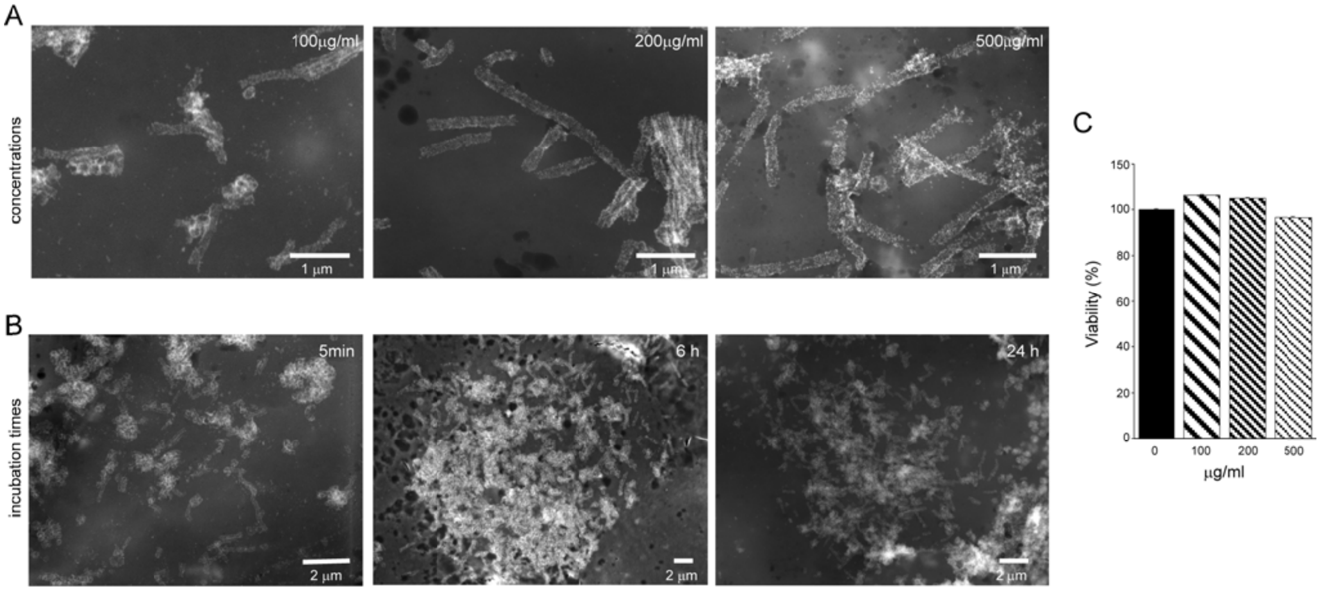

Supplementary Figure 2. ACNPs adhesion for different concentrations (A) and incubation times (B). Regardless of the concentration used (100, 200 and 500 μg/mL) and the length of incubation (5 min, 6 h and 24 h), ACNPs showed specific adhesion on microvilli. SEM BSE images. (C) Effects of CNPs on cell viability evaluated after 24 h incubation by MTT assay. Data are means of three independent experiments and expressed as a percentage of the control group.

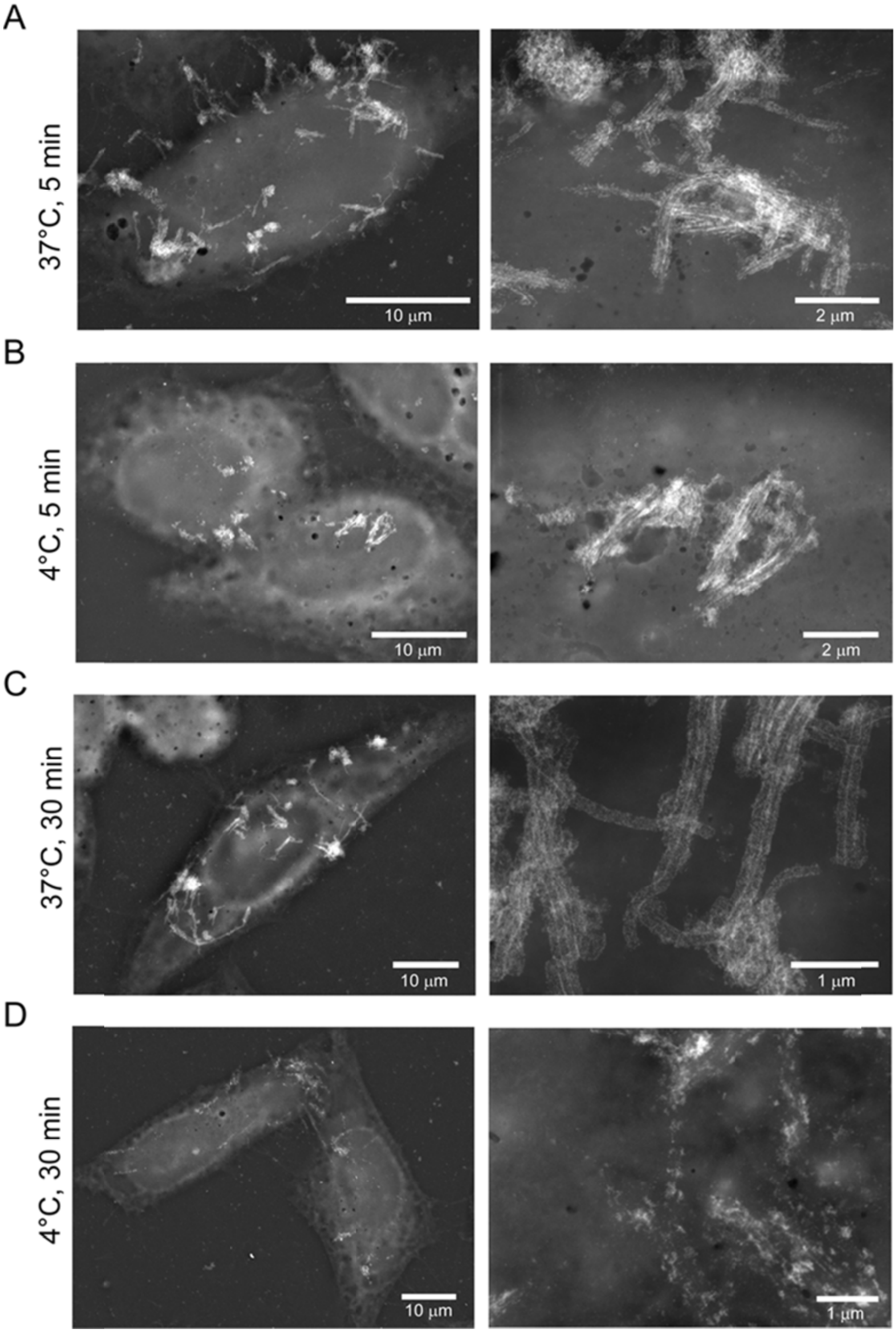

Supplementary Figure 3. ACNP adhesion was not blocked at low temperatures. After cells were kept for 30 min at 4°C, ACNPs were added to the medium and left for 5 (B) or 30 (D) min at 4°C. Cells incubated with ACNPs for 5 (A) or 30 (C) min at 37°C were the standard conditions. At 37°C, ACNP distribution appeared uniform along the microvilli, whereas at 4°C, the distribution appeared less homogeneous. HRSEM BSE images.

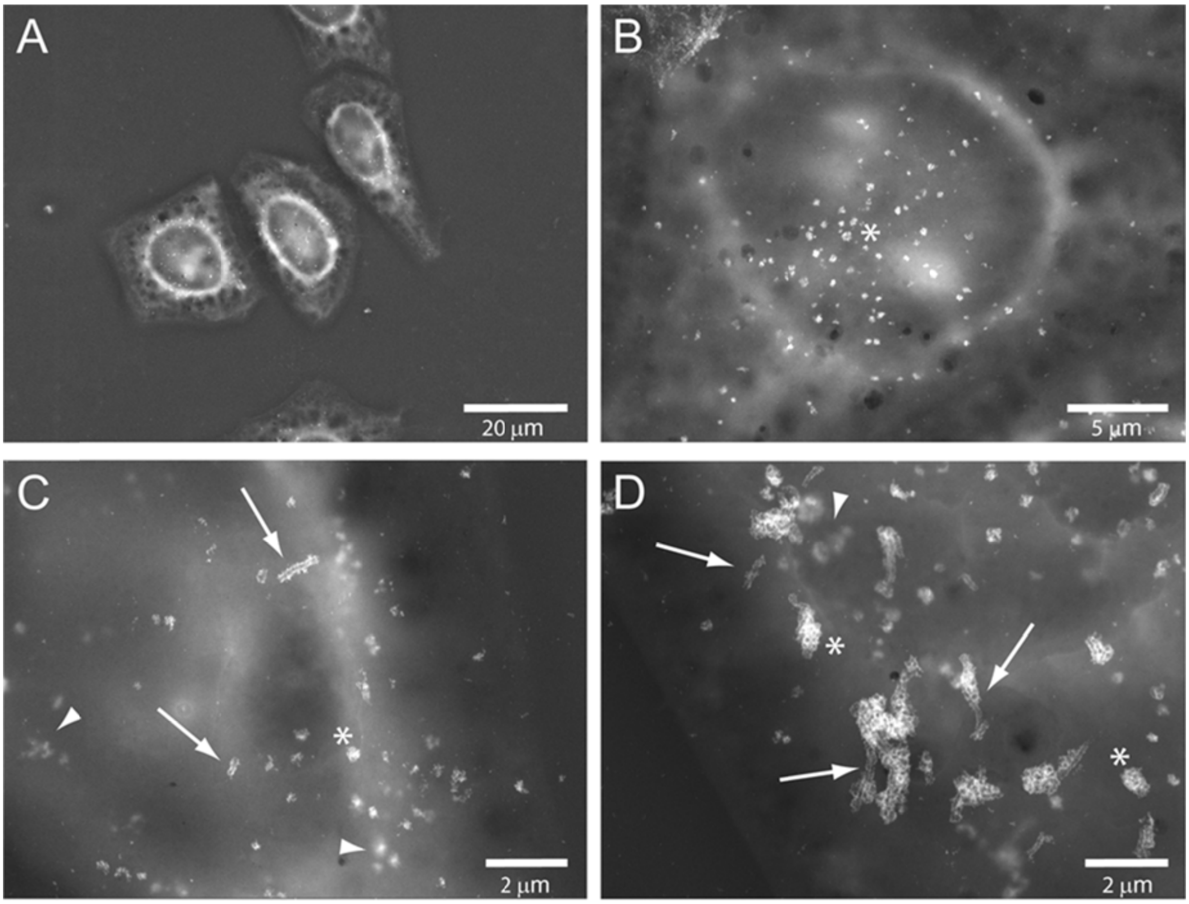

Supplementary Figure 4. HRSEM analysis of cells incubated with the ACNPs for 1 h (pulse) and fixed 2 h after complete removal of the particles (chase). (A) Cells at the end of 2-h chase. (B) Higher magnification of a single cell shows the presence of residual ACNPs. (C, D) ACNPs remained adhered to the microvilli (arrow) and/or bud-like structures (asterisk) on the cell surface. The presence of ACNPs on the cell surface, 2 h after their removal from the culture medium, suggests impairment of the dynamics of microvilli that have a physiological half-life of ~12 min. (D) Groups of ACNPs already internalized were also visible and appeared less bright (arrowhead).

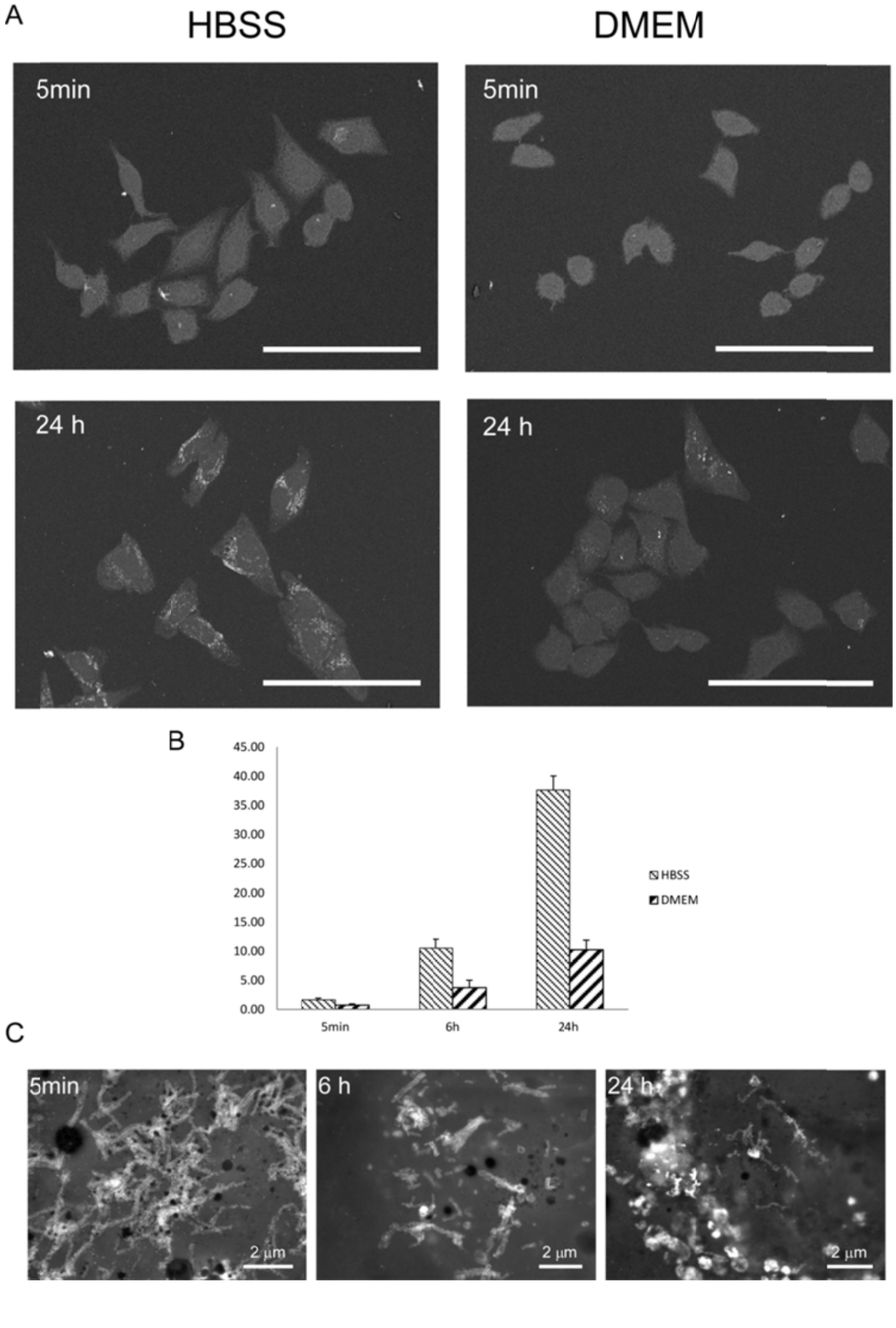

Supplementary Figure 5. ACNP adhesion was not mediated by proteins/components present in the culture medium. (A) Incubation of ACNPs in HBSS medium did not reduce cell adhesion compared to DMEM with FBS. HRSEM-BSE images. Bar = 50 µm. Images were acquired using an accelerating voltage of 15 kV with BSE detector. Under these conditions, both the ACNPs on the cell surface (brighter signal) and those already internalized (lower signal) could be observed. (B) After 5 min, 6 h and 24 h, more ACNPs interacted with cells when in HBSS than in DMEM. ACNP quantification was

obtained thresholding the images in order to include both signals. (C) ACNP distribution, when incubated with HBSS for different times. The interaction was similar to that observed with DMEM. The image showing ACNP distribution after 5 min incubation does not represent the average level of adhesion, but it was chosen to show the specificity of the interaction in the case of a cell particularly rich in microvilli. HRSEM-BSE images.

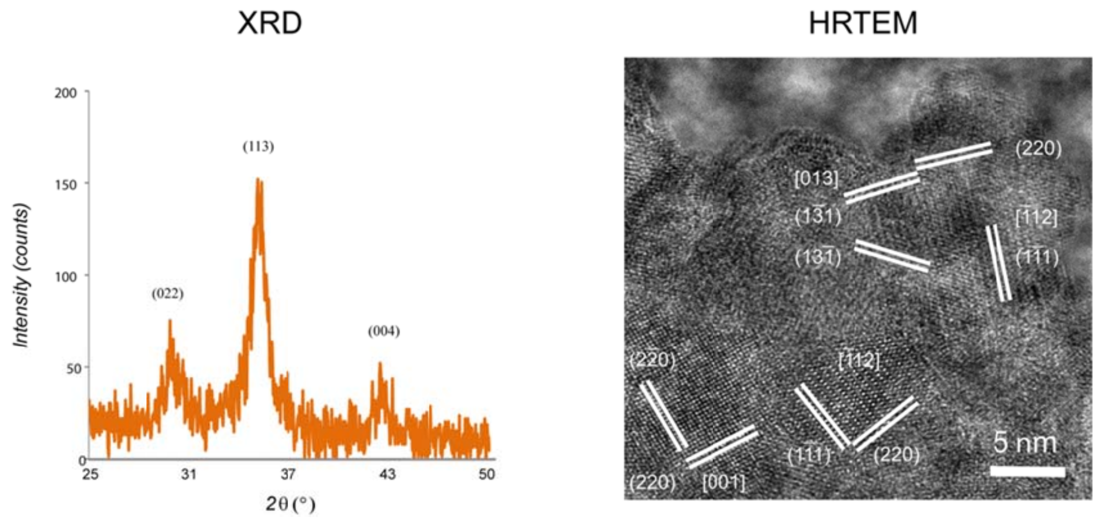

Supplementary Figure 6. XRD analysis and HRTEM imaging of IONPs.

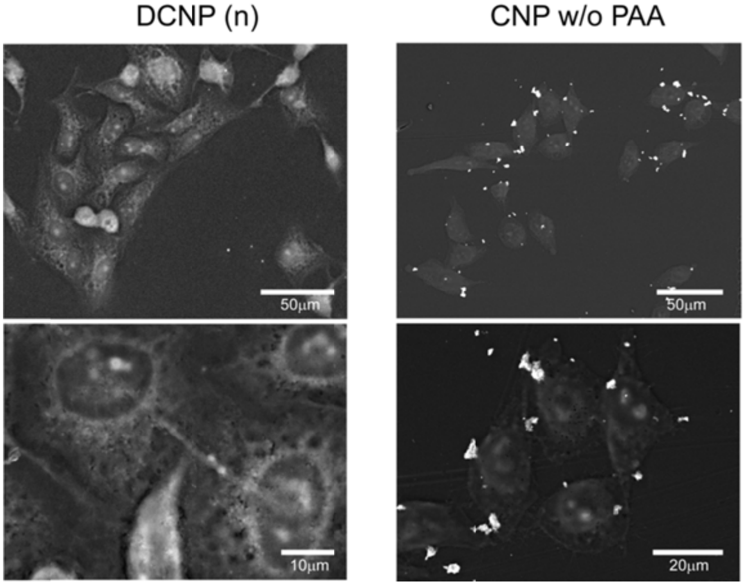

Supplementary Figure 7. CNP functionalization modified NP–cell interaction. Dextran coating reduced NP adhesion and internalization. Absence of PAA on the NP surface eliminated specific interaction with the cell membrane, resulting in random distribution of only a few large aggregates. HRSEM BSE images.
